# Classical Conditioning: Classical Yet Modern

**DOI:** 10.5334/pb.451

**Published:** 2018-07-26

**Authors:** Paul Eelen

**Keywords:** psychology of learning

## Abstract

This manuscript is part of a special issue to commemorate professor Paul Eelen, who passed away on August 21, 2016. Paul was a clinically oriented scientist, for whom learning principles (Pavlovian or operant) were more than salivary responses and lever presses. His expertise in learning psychology and his enthusiasm to translate this knowledge to clinical practice inspired many inside and outside academia. Several of his original writings were in the Dutch language. Instead of editing a special issue with contributions of colleagues and friends, we decided to translate a selection of his manuscripts to English to allow wide access to his original insights and opinions. Even though the manuscripts were written more than two decades ago, their content is surprisingly contemporary. The present manuscript was originally published as part of a Liber Amicorum for Paul Eelen’s own supervisor, prof. Joseph Nuttin. In this chapter, Paul Eelen presents a modern view on Pavlovian learning. It appeared in 1980, at the heyday of cognitive psychology which initially dismissed conditioning. Paul Eelen’s perseverance in presenting learning principles as key to study human behaviour has proven correct and ahead of time.

First published as: Eelen, P. (1980). Klassieke conditionering: Klassiek en toch modern. In Liber Amicorum, Prof. J. R. Nuttin, *Gedrag, dynamische relatie en betekeniswereld* (pp. 321–343). Leuven: Universitaire Pers Leuven.

Even though ever more complex areas of research have found their way into psychology, “Pavlov’s dog” continues to fascinate many researchers. What causes the enduring fascination with conditioning research? Does such research even have psychological significance? Would it not be better if it remained a study field for physiologists, as it originally was? The answers to these questions are partly determined by one’s conceptualization of classical conditioning. Most people are by now sufficiently familiar with its schematic representation: a *conditioned stimulus* (CS) elicits a *conditioned response* (CR), provided this stimulus has repeatedly been presented together with an *unconditioned stimulus* (US) that “inherently” elicits an *unconditioned response* (UR). Several limiting conditions qualify this schematic depiction. The CS must be “neutral” vis-à-vis the US. In other words, it cannot spontaneously elicit a response that is identical to the UR. The US must “inherently” elicit a well-defined response, which is why stimuli that are biologically significant for the studied organism are typically used (for some theorists, this became a necessary condition for conditioning to take place). The resulting CR must be an autonomous response that is part of the reaction pattern that the US evokes. This schematic depiction also provides insight into the necessary (and sufficient) conditions for conditioning to occur: both stimuli have to occur simultaneously. Finally, this schematic representation already implies what is learned: learning is equated with the modified reaction pattern vis-à-vis the CS. To put it simply, the dog learns to salivate at the sound of the bell.

This schematic depiction and the limiting conditions it implies constitute a strong simplification of the original phenomenon. After all, Pavlov’s interest in conditioning originated from his observation that the dog started to salivate when it heard and saw the man who brought the food. This rather complex event – someone who brings food – was ultimately reduced to a little lamp or an auditory signal predicting food. The “food” event of seeing a meat chunk in a bowl was reduced to the injection of meat powder directly into the animal’s mouth. The dog’s overall reaction pattern upon hearing the man who brings the food – and anyone who has a dog will be familiar with this pattern – was ultimately reduced to droplets of saliva (the reductive nature of this response was already highlighted by Zener, 1937). Moreover, the autonomous reaction that held Pavlov’s primary interest as a physiologist was initially not viewed as a core index of learning the relation between two events, but was subsequently seen as an almost integral part of the definition of classical conditioning (Gormezano & Kehoe, 1975). We can probably all concur with Rescorla and Holland’s related observation that “if conditioning were confined to what some have called “spit and twitches”, it would lose much of its psychological interest” (Rescorla & Holland, 1976, p. 184).

This strong reduction of the original events is probably characteristic of every type of operationalisation. This is justified in and of itself: operationalisations that reduce a phenomenon to its essence are vital for obtaining fundamental knowledge about the necessary and sufficient conditions that determine the occurrence of that phenomenon. But the danger exists that the question behind a concrete operationalisation is simply forgotten after a while. Moreover, there is a real danger that general statements and laws are formulated that are strongly connected to the concrete operationalisation. Something along those lines certainly happened in the study and appreciation of classical conditioning. The aim of this contribution, then, is to shed light on a number of recent trends in classical conditioning studies that might justify the title of this contribution. First, I summarize the most important findings that call for a broader framing of classical conditioning research. This is followed by a comprehensive discussion of one particular form of learning, that is, taste aversion that results from relations between the taste of food or drink on the one hand, and artificially induced nausea on the other hand. This phenomenon is sometimes referred to as the Garcia effect. The topic of taste aversion is discussed not because it is an almost prototypical example of classical conditioning, but because it contributed substantially to the questioning of important assumptions about conditioning. A number of authors have even called this the beginning of a “paradigmatic revolution” (Rozin, 1977; Bolles, 1975). The final part is somewhat speculative in nature: using the preceding observations as a starting point, it argues that a nontrivial similarity exists between recent theories in classical conditioning studies and those in a literature that at first glance appears to bear little relation to it, that is, attribution theories in social psychology.

## Classical conditioning: learning associations between two events

Every existing organism must in some way or another be sensitive to both meaningful as well as more coincidental relations between events in the environment, especially when such relations concern biologically significant events. At the same time, it would be maladaptive for an organism if the mere coincident occurrence of two events would be a sufficient condition for the organism to establish a connection between the two. Nevertheless, a coincident occurrence has often been considered a sufficient condition for learning a relation. When doubts were expressed about this idea, they concerned the nature of either one of both events (does one of the events need to have reinforcement value) rather than the nature of the relation itself (i.e., co-occurrence). What follows will demonstrate that every organism can process a wider range of informational relations than the mere joint occurrence of events. In describing this broad range, we aim to list general facts rather than to go deep into possible explanations.

### The role of contingency

**Figure 1:**
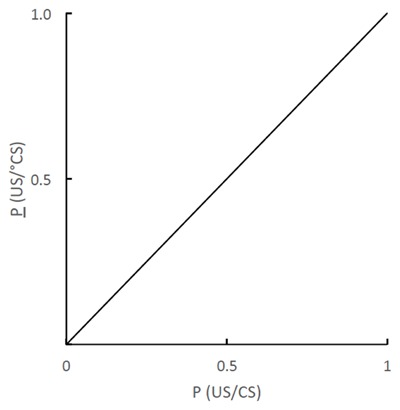
*Pavlovian contingency space*. The x-axis represents the conditional probability that the unconditioned stimulus (US) occurs together with the conditioned stimulus (CS). The y-axis represents the probability that the US occurs without the CS. There is no contingency between both stimuli on the diagonal line where both probabilities are equal (after Seligman, Maier and Solomon, 1971).

Instead of using terms indicating co-occurrence, relations can also be expressed in terms of correlation or contingency. What is emphasised in this case is not the temporal relation between two events but their logical relation. Applied to the situation of Pavlov’s dog, this means that a perfect positive correlation is introduced between the CS and the US in the experimental context. In other words, the conditional probability that the US is presented, given that the CS has been presented, equals 1; the probability that the US is presented in the absence of the CS equals 0. This is symbolically expressed as ρ(US/CS) = 1.0 and ρ(US/°CS) = 0. This can probably be further illustrated using what Seligman, Maier and Solomon called “The Pavlovian contingency space” (cf. Figure 1).

Given that the essence of the classical conditioning procedure lies in the experimenter’s full control over the two stimuli that are presented, this paradigm lends itself superbly to a study of the effects of variations in the correlational strength of CS-US relations.

Rescorla (1968) was one of the first to study this issue systematically. Over several experiments (Rescorla, 1975), he demonstrated that animals are sensitive to variations in contingency, ranging from a perfect positive correlation to a perfect negative correlation (respectively below and above the diagonal in Figure 1.) In this way, he succeeded in translating Pavlov’s two most important findings – excitatory and inhibitory conditioning – into contingency terms. Excitatory conditioning occurs whenever the animal learns that the CS and US tend to go together, in other words when ρ(US/CS) > ρ(US/°CS). A large number of behavioural indices then allow one to determine that the animal is behaving as if it “expects” the US when the CS is presented. Inhibitory conditioning occurs when the animal learns that the US and the CS tend not to go together, ρ(US/CS) < ρ(US/°CS). In this case, when the CS is presented, the animal will behave in a manner that is opposite to how it would behave in excitatory conditioning. When the CS and US are “randomly” presented, with no relation between both stimuli in other words, or ρ(US/CS) = ρ(US/°CS), it is observed that the CS does not acquire a new significance for the animal; in other words, the CS does not elicit a differential reaction. This nonetheless represents a form of active learning: learning that there is no relation is not synonymous to not learning (Mackintosh, 1973; Seligman, 1969). Let us illustrate this rather abstract formulation for what is known as “fear conditioning”, which is usually operationalised through the administration of an electrical shock as a US and an external stimulus (e.g., a visual signal) as a CS.

When, within the context of the experiment, the probability of a shock increases after the presentation of a given visual signal, this stimulus acquires a signalling function for the shock: the animal will behave “anxiously” when the CS is presented. If, however, the chance that a shock is administered is lower after the visual stimulus than in the absence of that stimulus, the animal will behave in a fairly “relaxed” fashion when the CS is administered. When the visual signal and the shock are “randomly” presented, the visual signal does not acquire a special meaning. Instead, the context as a whole becomes “fear-inducing” to the animal (Seligman, 1968).

The need for a contingent relation already indicates that mere co-occurrence is not a sufficient condition for an organism to learn the relation between two events. Some way or another, the organism is sensitive to the predictive value of stimuli and the covariance of events in its environment. What follows will illustrate that even a perfect contingency does not constitute a sufficient condition.

### Latent inhibition

Lubov (1973) coined the term “latent inhibition” to describe the following observation: when a stimulus is repeatedly presented by itself (i.e., without a US) in a particular context and when it is subsequently always followed by a US, it is difficult to obtain conditioning. It is not all too clear whether one should invoke non-associative or associative principles to explain this phenomenon. On the one hand, it could be argued that the organism no longer is attentive to the stimulus and, as it were, no longer even notices the stimulus because it has repeatedly been presented in the past. On the other hand, it could be argued that the organism has learned that the stimulus is irrelevant because the stimulus has repeatedly been presented on its own and that it afterwards struggles to realise that it is precisely this stimulus that should be considered the signal for an important event (Mackintosh, 1975). There is a certain similarity between latent inhibition and what occurs when a US is administered repeatedly before it is preceded by a CS. Here too, the results show that it is difficult to make this CS acquire the function of a signal for the US. For instance, when a series of shocks are administered in a non-contingent fashion and every shock is afterwards preceded by a tone in that same context, it takes a long time for the organism to learn this tone-shock relation. Again, the explanation for such data can be sought in non-associative or associative principles (Randich & Lolordo, 1979).

### Overshadowing

When two stimuli are presented together and consistently followed by a US, often only one of those stimuli will acquire the function of a signal for the US. Pavlov (1927) already discussed this phenomenon extensively and related it to the difference in “saliency” of the stimuli (as determined by the modality and intensity of the stimuli). Formal classical conditioning models have built in this “saliency” as a parameter – either as a fixed value (Rescorla & Wagner, 1972) or as a fluctuating value in accordance with the relation to the US (Mackintosh, 1975).

### Relative information value

Suppose that stimulus A and B are presented together and followed by an electric shock. In group I, stimulus B is also presented separately but not followed by a shock in between the A+B presentations. In group II, B is also presented separately every now and then, but here it is followed by a shock. In group III, only A+B trials are presented. The question is what happens to the signal value of stimulus A. A and a shock are after all paired an equal number of times in all groups. The relative information value of A, however, varies between groups because B is presented separately in groups I and II. In group I, A becomes the best predictor for a shock. B is a better predictor in group II, while the information value of both stimuli is equal in group III. When A is now separately tested in the three groups, conditioned responding varies in accordance with the manipulated information value (Wagner, 1969).

### Blocking

No phenomenon has probably made a larger contribution to clarifying the complexity of conditioning than blocking. It would be impossible to comprehensively list the relevant literature. We will therefore limit ourselves to a description of the basic phenomenon. Kamin (1969) was the first to bring this phenomenon to light in his “overshadowing” studies. Stimulus A (e.g., a visual signal) is frequently followed by an electric shock. When the conditioning is complete, stimulus B (e.g., an auditory signal) is presented together with stimulus A, and both are followed by a shock. B does not acquire a signal value even though there is a perfect correlation between B and a shock from this moment onward. This is evident from the fact that when B is presented on its own, it does not elicit a response. It is as if the previous conditioning of A is blocking the conditioning of B, hence the term. There are indications that the animal does notice stimulus B, but that it learns as it were that B presents irrelevant or at least redundant information about the US (Mackintosh, 1978).

### Conclusion

The above information clearly indicates that mere stimulus co-occurrence is not a sufficient condition for an organism to relate two events. The discussion below will demonstrate that it is also not a necessary condition, which again offers a different perspective on classical conditioning. Instead of an automatic process that plays out in a passive organism, the organism emerges as an active information-processing system.

It is probably possible to relate all the phenomena that were discussed above to the role of contingency. But the question remains what mechanism can be invoked for explaining the role of contingency. Some do not hesitate to postulate that the animal has a cognitive representation of the contingency space (Alloy & Seligman, 1979). Others have drawn more cautious conclusions. As Rescorla notes:

*“Most of us are not comfortable with the notion that organisms take in large blocks of time, count up numbers of US events, and somehow arrive at probability estimates … It is tempting to think of simple “tricks” that the organism could use to perform in this apparently rational fashion” (Rescorla, 1969, p. 84–85)*.

In other words, being influenced by a correlational relation does not ipso facto imply that the organism concerned has any understanding of this correlation. It is therefore remarkable that Rescorla, who perhaps highlighted the role of contingency more than anyone else, succeeded in developing a theory in which the learning of relations can be traced back to the co-occurrence of two events after all (Rescorla & Wagner, 1972). At the level of formalisation, this theory remains purely descriptive. We would like to note, however, the psychological intuition on which it was built (Rescorla, 1969). The notion of “expectation discrepancy” is central here. As soon as something (important) happens unexpectedly – in other words, it was not predicted – it is as if the animal starts searching for a predictor for this event. Expectation discrepancy appears to be a necessary condition for a stimulus to be interpreted as the signal for this unexpected event. No new learning occurs when either the context (see latent inhibition) or other signals (see blocking) had already predicted the event. This “expectation discrepancy” also explains inhibitory conditioning: when an event that an organism expects to occur in a particular context does not occur, a stimulus that is correlated with this expectation discrepancy may acquire an inhibitory function. Note that this theory emphasizes the role of the environment and the organism’s prior history. We deliberately use metaphors like “to start searching for a predictor”, “to interpret an event” etc. It is as if the facts can only be described in such terms. Such language becomes even more imperative when describing taste aversion.

## Taste aversion: The Garcia effect

A short article by Garcia and Koelling published in *Psychonomic Science* in 1966 was the starting point of the literature on what is now known as the Garcia effect. At the time, there was nothing to suggest that the article would become a classic. Quite the contrary, the article had been rejected by a more renowned journal, which the then editor would later express his regrets about. As is often the case with “classics”, the article was indeed rather weak at the methodological level, but it contained fairly far-reaching theoretical implications. Today, these are referred to as the “Garcia effect”, “the message of Garcia” and “the paradigmatic revolution”. At least 600 articles that were more or less inspired by the Garcia effect have been published since then. This exceptional level of attention does not guarantee scientific relevance in itself. Garcia’s findings may have originally been called into question due to their methodological shortcomings, but the extensive attention has at the least ensured sufficient subsequent independent replications of Garcia’s experiments. The phenomenon is real. The debate about its reach and interpretation, however, remains active today. We first discuss the meaning of “the message of Garcia” by describing a couple of typical experiments. We subsequently reflect on the varying attempts that have been made to interpret this phenomenon.

### “The message of Garcia”

What the message of Garcia essentially revolves around is probably best illustrated with an anecdote recounted by Seligman (Seligman & Hager, 1972). After he was served “filet mignon with béarnaise sauce” during a dinner, he became unwell at night. This nausea later proved to be a harbinger of a flu attack. But Seligman had already ascribed it to the béarnaise sauce, and since then he cannot suffer the look, let alone the taste of this sauce. This anecdote raises several questions. Why did he “ascribe” his becoming sick to the béarnaise sauce? Why not to the filet, the dessert or the drinks? Why not to the restaurant or the other guests? Why did his aversion to béarnaise sauce not disappear when it later turned out that the flu was a far more likely cause? Why did béarnaise sauce taste so bad since then? It turns out that answering these questions becomes difficult when this event is translated into a conditioning paradigm, with flavour as the CS and becoming sick as the US (or UR). The most noticeable departures from the normal rules are the extended time period between the CS and US and the difficulty of the extinction, even after the adjusted interpretation. The nature of the US moreover appeared to determine the selection of the CS and, finally, a process that is qualitatively different appears to be at stake here: the béarnaise sauce is avoided not because it is seen as a predictor of nausea, but because it acquires an intrinsically bad flavour.

Garcia’s studies evoke similar questions. The discussion of a typical study will illustrate this further. Garcia and Koelling (1966) deprived caged rats of water for the duration of the experiment. Every day, the rats were placed in individual test cages that contained a drink tube. After an adjustment period in which clean water was offered, the learning phase began. The water was replaced by a saline solution. Every time the drink tube opening was touched, a visual and auditory stimulus were presented so that every drinking attempt was paired with a “bright-noisy-tasty” constellation of stimuli. In the first group, drinking coincided with a period of radiation (X-rays).^1^ In the second group, lithium chloride was used as the saline solution; this has a poisonous effect but the rats cannot distinguish it from a non-poisonous saline solution (Nachman, 1963). In a third group, an electric shock was administered two seconds after drinking. This learning phase was spread over several days in all the groups. On non-conditioning days, the test cage contained only normal water, the drinking of which was not paired with the abovementioned constellation of stimuli. This was followed by a test phase in which either the audiovisual stimulus or the flavour (saline solution) without the audiovisual stimulus was presented during the drinking of clean water. In the X-ray and lithium groups, there was a clear suppression of drinking with the flavour test but not with the audiovisual test, while precisely the opposite occurred in the shock group. In other words, there appears to be an interaction between the nature of the discriminative stimulus and the drinking consequences.

At first glance, several findings regarding conditioned taste aversion indeed contradicted the basic rules of conditioning. First and foremost, there was a clear parametric difference with more typical conditioning preparations: the time interval between the taste CS and administration of the aversive US (induced sickness) was typically much larger. “Records” of 24 hours of difference were set (Etscorn & Stephens, 1973)! In the experiment described above, an interaction moreover exists between the nature of the CS and the nature of the US, which is probably the finding that has prompted the most discussion: It is not possible to learn an association between whichever two things. Finally, we already noted that we are seemingly dealing with a qualitatively different phenomenon.

### Theoretical reflections

The different attempts to explain flavour aversion can be separated into two main orientations. A first orientation refers to the biological nature of every organism. Through the course of natural selection, every organism has come to be equipped with specific learning mechanisms that, depending on the organism’s adaptation, show specific characteristics as a function of the different challenges the animal faces in its environment. For instance, it is indeed vitally important for an animal to learn the association between certain food attributes and certain metabolic effects. A second orientation attempts to reconcile the properties of flavour aversion with the more general fundamental rules that govern the learning of relations between two events. It does not deny that parametric and perhaps qualitative differences clearly exist between learned taste aversion and the more conventional conditioning findings. But these differences supposedly originate from the particular characteristics of the used stimuli. Insofar that these characteristics can be described, their influence can be assessed through experiments – independent of the flavour aversion phenomenon.

It is indeed remarkable that all the factors that influence learning of an association between two events (cf. below) also have an influence on learned flavour aversion. First, there is the impact of contingency. Inducing a flavour aversion requires a positive correlation: “random” administration of a flavour and US does not have an effect, and a negative correlation between a flavour and the US results in a preference for this flavour (Best, 1975). Latent inhibition is also possible: flavour aversion is slow to develop when the animal is made to taste a certain flavour repeatedly before it is paired with an aversive substance (Domjan, 1972; Elkins, 1973). It is equally clear that when the animal is first repeatedly made ill in a way that is non-contingent to ingestion of a particular food, the animal subsequently no longer ascribes this becoming sick to the flavour of the food (Braveman, 1977). “Blocking” finally has also been demonstrated; a learned aversion to a particular flavour can “block” learning of aversion to a different flavour (Revusky, 1971). It is important to note a study by Rudy, Iwens and Best (1977) in this regard. They first induced a contingency between an external stimulus (black cage) and nausea. When the flavour of saccharine was subsequently involved in this contingency, the animal no longer ascribed the nausea to the flavour. This study is important in two regards. First, it demonstrates that associations between external stimuli and “nausea” can indeed be learned as long as an external stimulus is used that is fairly salient and that can compete with a flavour stimulus in terms of “novelty”. In addition, the results of this experiment certainly do not appear to correspond to what one would expect from a “preparedness” view. If learning of a flavour-nausea contingency is truly “prepared”, it does not seem very plausible that learning of this contingency can be fairly easily “blocked” by a pre-induced artificial or at least unprepared contingency.

These findings indicate that the Garcia effect is not as extraordinary as it appears to be at first glance and that it can in fact be integrated into the more general findings about association learning (Logue, 1979). The particular characteristics of the Garcia effect, however, have urged reflection on the more conventional procedures from a different perspective.

Consider, for instance, the parametric difference between flavour aversion and set-ups that are more conventional in terms of the time lapse between the CS and US (or between the discriminative stimulus and reinforcement). The hypothesis of an after-flavour during nausea was of course the most simple one, but it was emphatically rejected empirically (Revusky & Garcia, 1970). It is therefore almost certain that the Garcia effect is due to a memory phenomenon. When the rat becomes sick, he “remembers” the type of food that may have caused this. Revusky (1971, 1977) integrates these findings into what he describes as a more general associative interference theory. This theory inspired Lett (1973, 1974, 1975, 1977) to demonstrate that a rat is capable of bridging a fairly large time interval between a discriminative stimulus and reinforcement – and this with more conventional procedures. For this to happen, however, the situation must be designed so that the animal is urged to again “call to mind” the discriminative stimulus during reinforcement. It is remarkable that the Garcia effect, which is so deeply rooted in the biological singularity of the organism, is an illustration of the animal’s cognitive capabilities and that it has helped integrate recent findings in the psychology of memory into conditioning studies (Best & Gemberling, 1977; Wagner, 1978).

In addition, there is the interaction between the nature of the CS and the US. This interaction is in fact only exceptional when one merely considers the external characteristics of a relationship between two events (contiguity, contingency). It becomes more comprehensible when one assumes that other factors also exist that influence the learning of relations, such as similarity, spatial factors, etc. This insight was probably best articulated by Testa (1975, 1976); he related learned flavour aversion to the more general question of how the animal perceives causal relations in its natural environment. He argues for factors to be integrated into the study of conditioning that had already been previously underlined by gestalt psychologists in relation to perception. We find a similar plea in Revusky (1977) and Rescorla and Cunningham (1979).

## Conditioning and attribution

After demonstrating how an organism can process complex relations, we have presented a discussion of learned flavour aversion because the latter highlights a central problem: what pushes the animal to selectively attribute certain effects to the ingestion of food or drink when both events are so far removed in time? Rather than viewing this simply as an innate mechanism, it was argued that this phenomenon should be integrated as much as possible into what we know about the learning of associations between events. What then is the meaning of all of this? It seems that there is a fundamental similarity between these findings in the conditioning literature and attribution theory as it was developed in the social psychological literature. This observation suggests that common principles exist that cause both humans and animals to discover causal relations in their environment. Such a speculative observation probably requires a number of prior explanations. Attribution theory is the study of the manner in which certain events are explained in terms of their potential causes. Born from the field of social psychology – a historic coincidence in Kelley’s view (1967) – the main topic of attribution research was people’s causal analysis of the behaviour of others and one’s self. In other words, on the basis of which rules do I infer the “why” behind my own or other people’s actions? But in essence, a much broader question is at stake in attribution theory: how does one make causal inferences between all sorts of events? The question even arises whether a clear distinction ought to be made between a causal interpretation of events and a causal interpretation of actions. The distinction between “cause” and “reason” is key here, and it was also the focus of a recent discussion (Buss, 1978; Harvey & Tucker, 1979; Kruglanski, 1979; Buss, 1979). Only causal interpretations of events will be discussed below. To put it in more trivial terms, when a rat is administered a shock by the experimenter, it might ask itself: What is this shock due to (asks after the cause)? It does not ask: why did this experimenter give me a shock (asks after the reason)? Both are “why” questions, but they are logically different from each other. Second, a distinction should be made between the attribution *process* and the *content* of the attributions (Nisbett & Wilson, 1977; Kruglanski, 1979). At the level of contents, it is obvious that any animal-human comparison would be a tenuous one. But this is also true for a comparison between mutual humans, if only because of cultural differences (Kruglanski, 1979). As regards attribution as process, it is probably possible to arrive at more general statements about the heuristics that apply to both humans and animals. A third introductory remark concerns the status of the concept of attribution. Attribution is intended as a “mediating” concept that can either be assigned a reality value or an “as if” nature. This is true for most “cognitive” concepts that were designed to mediate between input and output (consider, for instance, the concept of “expectancy”). In our view, there is a trend towards increasing emphasis on the “as if” nature of attributions in social psychology. The most common descriptions – somewhat schematically – present this sequence as follows: 1. something occurs (S) – 2. the organism asks itself “why” – 3. following deliberation, it arrives or does not arrive at a judgement – 4. it acts in a manner that is consistent with this (R). If assigned a reality value, it is possible to render the typically non-observable links 2. and 3. observable in humans by simply inquiring after them. It would not be exaggerated to state that this is the focus of most attribution research. And any study of attributions in animals is of course impossible in this respect. But it has been asked more and more whether the “links” do not acquire a different status precisely because they are made observable. Let us again briefly go back to Seligman’s anecdote about the béarnaise sauce. If Seligman is asked: “Why did you become sick?”, he will answer: “Because I had a flu attack.” In other words, does a “conscious” reflection on the occurrence of an event not respond to different rules than the total original experience of this occurrence? Is this not where the truth lies of Pascal’s statement that “le cœur a ces raisons que la raison ne connaît pas”? In a rather extensive article, Nisbett and Wilson (1977) defended the proposition that these cognitive mediating processes circumvent every form of introspection. To support their argument, they cited a number of statements by cognitive psychologists, including Neisser and Mandler, that we would like to cite here. For instance, Neisser writes that “the constructive processes (of encoding perceptual sensations) themselves never appear in consciousness, their products do” (Neisser, 1967, p. 301). And Mandler considers that “there are many systems that cannot be brought into consciousness, and probably most systems that analyze the environment in the first place have that characteristic. In most of these cases, only the products of cognitive and mental activities are available to consciousness” (Mandler, 1975, p. 245). Although Nisbett and Wilson’s proposition is debatable (Smith & Miller, 1978), Langer (1978) does not hesitate to go one step further: she simply denies the mediating role of conscious cognitions in most of our day-to-day actions: “Much psychological research relies on a theoretical model that depicts the individual as one who is cognitively aware most of the time, and who consciously, constantly, and systematically applies “rules” to incoming information about the environment in order to formulate interpretations and courses of actions. Attribution theorists rely on this model in attempting to uncover the sources of regularities in human behaviour. But if in fact it can be demonstrated that much complex human behaviour can and does occur without these assumed cognitive assessments, then we must question both pervasiveness of attribution making as a cognitive process and the assumptions made by most social psychologists” (Langer, 1978, p. 35). We find a similar plea to look for very simple heuristics to explain the notion of attribution in Kahneman and Tversky (1973), Pryor and Kriss (1977) and especially Taylor and Fiske (1978) who concluded that most attribution processes “seem to occur automatically and substantially without awareness, and as such, they differ qualitatively from the intentional, conscious, controlled kind of search which we like to think characterises all our behaviour” (Taylor & Fiske, 1978, p. 283).

**Figure 2:**
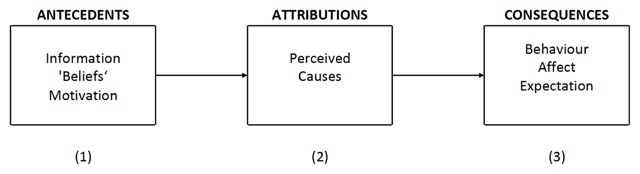
Schematic model of attribution research (after Kelley & Michela, 1980).

These introductory explanations probably create more room for the proposition that there is something common about the way that humans and animals infer causal relations. We rely on a recent overview article by Kelley and Michela (1980) on attribution theory to buttress this claim in a more direct way. They first offer the general scheme that is implicitly contained in the study field of attribution (Figure 2).

As indicated above, any type of direct research into (2) is evidently impossible with animals. One has to limit oneself to manipulations of (1) and inferring what happens in (2) from a change in (3). But as also noted above, this limitation probably also applies to studies of attribution in humans. On the antecedent side (1) then, there is a clear similarity between the factors influencing the nature of attributions and conditioning. Let us illustrate this using the principles that Kelley and Michela distilled from attribution literature. These principles hold that certain aspects of the information that the organism is confronted with lead to attributions. Almost every one of these is a principle that we already mentioned in our discussion of the factors that influence conditioning.

- *Covariance*: The ANOVA model. This principle was primarily emphasised by Kelley himself (Kelley, 1967, 1973). “The effect is attributed to that condition which is present when the effect is present and which is absent when the effect is absent” (Kelley, 1967, p. 194). This covariance principle is of course heavily analogous, if not identical, to the role of contingency in classical conditioning. This raises a twofold observation: first, there is no reason to suppose, as Kelley does, that the influence of this covariance principle must revert to a model of the human as a “naïve” scientist who thinks according to an ANOVA model (he probably only does so in the context of attribution experiments!). As noted above, the influence of contingency does not necessarily imply that the organism has any notion of contingency. In addition, there is the dilemma of moving from a correlation judgement to a causal judgement. A causal relation after all implies a correlation, but the reverse does not hold. Is a causal judgement only possible when one implicitly also has knowledge of the mechanisms that connect cause and effect? Or are other conditions necessary in addition to perfect correlation for two events to be perceived in a cause-effect relation? This dilemma brings us to the question posed by Michotte (1954): Is causality a phenomenal experience or a “post hoc” reflection? It is interesting to note in this regard that Testa (1974) relied on Michotte’s findings to explain flavour aversion.
- *Saliency*: “The notion here is that an effect is attributed to the cause that is most salient in the perceptual field at the time the effect is observed.” (Kelley & Michela, 1980, p. 466). This “saliency” is again a factor that plays a role in conditioning (see below).
- *Similarity and Contiguity*: The principle of contiguity does not require much explanation to be related to conditioning. Rescorla and Furrow (1977) convincingly demonstrated the role of “similarity”, which has always been seen as an associative principle, within a conditioning paradigm.
- *Primacy*: “The general notion here is that a person scans and interprets a sequence of information until he attains an attribution from it and then disregard later information or assimilates it to his earlier impression” (Kelley & Michela, 1980, p. 467). Conditioning literature analogies also exist for this. It for instance takes a long time for the animal to recognise a “random” relationship such as when a tone and a shock are “randomly” presented but this random series begun with a contingency between both events. The reverse is also true: a contingency is also learned with difficulty in the case of a random start and subsequent contingency (Alloy & Seligman, 1979).

Conditioning literature parallels also exist for the interaction between the nature of the information on the one hand and the existing “beliefs” or causal models and the motivational component (Figure 2) on the other. The “blocking” phenomenon can be considered a causal model that interferes with the learning of other causal relations: both humans and animals do not look for every possible cause but instead suffice themselves with one sufficient cause. In addition, the motivational component has always been central to conditioning. To explain this using anthropomorphic terms, the animal only asks itself a why-question when something important occurs.

We here touch on a point that made us relate the notion of attribution to findings on conditioning. We prefer the term attribution over the term association to denote what happens during conditioning. Not only because “association” is a historically heavily charged concept, but because the term does not permit a distinction between the propositions “event X reminds me of event Y” and “I ascribe event X to event Y”. To again illustrate this using Seligman’s example: when he becomes nauseous, he can perfectly remember the full dinner event, but only one relation, one “attribution” is made with the béarnaise sauce. Remembrance is a necessary but not a sufficient condition to establish a causal relation between two events. Winograd (1971) described this as follows: “Let us imagine that I emerge from my house in the morning and find a flat tire on my car. It occurs to me immediately that around nine o’clock the previous evening, while driving home, I heard a disturbingly loud noise as I drove over something in the road. Now, 12 hours later, I “associate” the flat tire with the impact. This is not an association in the usual S-R contiguity sense; rather, I have related two events which were separated by a long period of time. I can do this only if I have a record of the earlier event, or memory. In fact, I have many memories of previous events, and that is the problem. The question to be dealt with is one of trace selection or contact, of how I have related these two particular events” (Winograd, 1971, p. 272–273). This, precisely, is the dilemma posed by the Garcia effect, looked at from a different perspective. It is why use of the term “attribution” rather than the term “association” becomes even more imperative when we keep the phenomenon of flavour aversion in mind. As Revusky and Garcia write, “Probably, the rat can really associate these events, but will not attribute the production of shock to the flavored water. In other words, a rat can learn that consumption of flavored water *precedes* shock, but will not readily learn that consumption of flavoured water *produces* shock” (Revusky & Garcia, 1970, p. 41). A bit further, both authors write: “This paper would probably be more precise if, whenever the term “association” is used, “attribution” were to be substituted” (p. 43). Does, after this discussion, it still seems absurd that an animal responds “as if” it were making an attribution?

## Conclusion

Of course, a change in terminology is only a pseudo-solution to a dilemma that has been key since Pavlov’s dog: How is a relation between two events learned? This contribution offers only limited insight into this question, and it is quite fortunate that the effective learning of such relations does not depend on its explanation. But the search for such an explanation becomes imperative the moment it is established that the learning of relations fails. Because this probably constitutes a true breeding ground for human and animal suffering: the inability to explain an important event.

Finally, this contribution might foster the impression that contemporary conditioning psychology tries to anthropomorphise the rat too much, when in the past humans were seen too much as rats. But a rat is a rat and a human a human. Nevertheless, it does not seem very fruitful to me to hermetically seal off both study domains. Whereas Estes notes that “the thought arises that the processes and mechanisms of human cognition represent specializations and elaborations of processes and mechanisms which can advantageously be studied in animals that learn as well as in machines that think” (Estes, 1975, p. 6), this contribution was written from the conviction that Estes’ first alternative continues to be valuable. For as long as a computer does not salivate upon seeing a chunk of meat, “Pavlov’s dog” continues to be a fascinating phenomenon.

## Footnotes

The use of X-rays in this experiment probably calls for some explanation about the prior history of the Garcia effect. Commissioned by the ministry of defence, Garcia and his collaborators completed a series of investigations into the influence of ionising radiation on animal behaviour during the fifties. Their most important finding was that such radiation – even when administered in small doses – had an aversive nature to the animal, and the behavioural component that this could most clearly be inferred from was the strongly reduced drink and food intake. It proved much more difficult, however, to use these radiation effects to teach spatial avoidance behaviour (Garcia, Kimeldorf & Hunt, 1961).

## References

[1] Alloy, L. B., & Seligman, M. E. P. (1979). On the cognitive component of learned helplessness In: Bower, G. H. (ed.), The psychology of learning and motivation, 13 New York: Academic Press.

[2] Best, M. R. (1975). Conditioned and latent inhibition in taste-aversion learning: Clarifying the role of learned safety. Journal of Experimental Psychology: Animal Behavior Processes, 1, 97–113. DOI: 10.1037/0097-7403.1.2.97 1141823

[3] Best, M. R., & Gemberling, G. A. (1977). Role of short-term processes in the conditioned stimulus pre-exposure effect and the delay of reinforcement gradient in long-delay taste aversion learning. Journal of Experimental Pyschology: Animal Behaviour Processes, 3, 253–263. DOI: 10.1037/0097-7403.3.3.253

[4] Bolles, R. C. (1975). Learning, motivation and cognition In: Estes, W. K. (ed.), Handbook of learning and cognitive processes, 1 Hillsdale, N.J.: Erlbaum.

[5] Braveman, N. S. (1977). What studies on pre-exposure to pharmalogical agents tell us about the nature of the aversion-inducing treatment In: Barker, L. M., Best, M. R., & Domjan, M. (eds.), Learning mechanisms in food selection. Waco, Texas: Baylor University Press.

[6] Buss, A. R. (1978). Causes and reasons in attribution theory: A conceptual critique. Journal of Personality and Social Psychology, 36, 1311–1321. DOI: 10.1037/0022-3514.36.11.1311

[7] Buss, A. R. (1979). On the relationship between causes and reasons. Journal of Personality and Social Psychology, 37, 1458–1461. DOI: 10.1037/0022-3514.37.9.1458

[8] Domjan, M. (1972). CS pre-exposure in taste-aversion learning: Effects of deprivation and pre-exposure duration. Learning and Motivation, 3, 389–402. DOI: 10.1016/0023-9690(72)90002-1

[9] Elkins, R. L. (1973). Attenuation of drug-induced bait shyness to a palatable solution as an increasing function of its availability prior to conditioning. Behavioral Biology, 9, 221–226. DOI: 10.1016/S0091-6773(73)80156-7 4721216

[10] Estes, W. K. (1975). Introduction to Volume 2 In: Estes, W. K. (ed.), Handbook of learning and cognitive processes, 2 Hillsdale, N.J.: Erlbaum.

[11] Etscorn, F., & Stephens, R. (1973). Establishment of conditioned taste aversion with a 24-hour CS-US interval. Physiological Psychology, 1, 251–253. DOI: 10.3758/BF03326916

[12] Garcia, J., Kimeldorf, D. J., & Hunt, E. L. (1961). The use of ionizing radiation as a motivating stimulus. Psychological Review, 68, 383–395. DOI: 10.1037/h0038361 13896809

[13] Garcia, J., & Koelling, R. A. (1966). Relation of cue to consequence in avoidance learning. Psychonomic Science, 4, 123–124. DOI: 10.3758/BF03342209

[14] Gormezano, I., & Kehoe, E. J. (1975). Classical conditioning: Some methodological and conceptual issues In: Estes, W. K. (ed.), Handbook of learning and cognitive processes, 2 Hillsdale, N.J.: Erlbaum.

[15] Harvey, J. H., & Tucker, J. A. (1979). On problems with the cause-reason distinction in attribution theory. Journal of Personality and Social Psychology, 37, 1441–1446. DOI: 10.1037/0022-3514.37.9.1441

[16] Kahneman, D., & Tversky, A. (1973). On the psychology of prediction. Psychological Review, 80, 237–251. DOI: 10.1037/h0034747

[17] Kamin, L. J. (1969). Predictability, surprise, attention and conditioning In: Campbell, B., & Church, R. (eds.), Punishment and aversive behavior. New York: Appleton.

[18] Kelley, H. H. (1967). Attribution theory in social psychology In: Levine, D. (ed.), Nebraska symposium on motivation, 15 Lincoln: University of Nebraska Press.

[19] Kelley, H. H. (1973). The processes of causal attribution. American Psychologist, 28, 107–127. DOI: 10.1037/h0034225

[20] Kelley, H. H., & Michela, J. L. (1980). Attribution theory and research In: Rosenzweig, M. R., & Porter, L. W. (eds.), Annual Review of Psychology, 31 Palo Alto: Annual Reviews Inc DOI: 10.1146/annurev.ps.31.020180.002325 20809783

[21] Kruglanski, A. W. (1979). Causal explanation, teleological explanation: On radical particularism in attribution theory. Journal of Personality and Social Psychology, 37, 1447–1457. DOI: 10.1037/0022-3514.37.9.1447

[22] Langer, E. J. (1978). Rethinking the role of thought in social interaction In: Harvey, J. N., Ickes, W. I., & Kidd, R. F. (eds.), New directions in attribution research, 2 Hillsdale, N.J.: Erlbaum.

[23] Lett, B. T. (1973). Delayed reward learning: Disproof of the traditional theory. Learning and Motivation, 4, 237–246. DOI: 10.1016/0023-9690(73)90013-1

[24] Lett, B. T. (1974). Visual discrimination learning with a 1-minute delay of reward. Learning and Motivation, 5, 174–181. DOI: 10.1016/0023-9690(74)90024-1

[25] Lett, B. T. (1975). Long delay learning in the T-maze. Learning and Motivation, 6, 80–90. DOI: 10.1016/0023-9690(75)90036-3

[26] Lett, B. T. (1977). Long delay learning in the T-maze: Effects of reward given in the home cage. Bulletin of the Psychonomic Society, 10, 211–214. DOI: 10.3758/BF03329327

[27] Logue, A. W. (1979). Taste aversion and the generality of the laws of learning. Psychological Bulletin, 86, 276–296. DOI: 10.1037/0033-2909.86.2.276

[28] Mackintosh, N. J. (1973). Stimulus selection: Learning to ignore stimuli that predict no change in reinforcement In: Hinde, R. A., & Stevenson-Hinde, J. (eds.), Constraints on learning. London: Academic Press.

[29] Mackintosh, N. J. (1975). A theory of attention: Variations in the associability of stimuli with reinforcement. Psychological Review, 82, 276–293. DOI: 10.1037/h0076778

[30] Mackintosh, N. J. (1978). Cognitive or associative theories of conditioning: Implications of an analysis of blocking In: Hulse, S. H., Fowler, H., & Honig, W. K. (eds.), Cognitive processes in animal behavior. Hillsdale, N.J.: Erlbaum.

[31] Mandler, G. (1975). Mind and emotion. New York: Wiley.

[32] Michotte, A. (1954). La perception de Ia causalité. Louvain: Studia Psychologica.

[33] Nachman, M. (1963). Learned aversion to the taste of lithium chloride and generalization to other salts. Journal of Comparative and Physiological Psychology, 56, 343–349. DOI: 10.1037/h0046484 13937024

[34] Nisbett, R. E., & Wilson, T. D. (1977). Telling more than we can know: Verbal reports on mental processes. Psychological Review, 84, 231–259. DOI: 10.1037/0033-295X.84.3.231

[35] Pavlov, I. P. (1927). Conditioned reflexes. London: Oxford University Press.

[36] Pryor, J. B., & Kriss, M. (1977). The cognitive dynamics of salience in the attribution process. Journal of Personality and Social Psychology, 35, 49–55. DOI: 10.1037/0022-3514.35.1.49

[37] Randich, A., & LoLordo, V. M. (1979). Associative and nonassociative theories of the UCS pre-exposure phenomenon: Implications for Pavlovian conditioning. Psychological Bulletin, 86, 523–548. DOI: 10.1037/0033-2909.86.3.523 377357

[38] Rescorla, R. A. (1968). Probability of shock in the presence and absence of the CS in fear conditioning. Journal of Comparative and Physiological Psychology, 66, 1–5. DOI: 10.1037/h0025984 5672628

[39] Rescorla, R. A. (1969). Conditioned inhibition of fear In: Mackintosh, N. J., & Honig, W. K. (eds.), Fundamental issues in associative learning. Halifax: Dalhousie University Press.

[40] Rescorla, R. A. (1975). Pavlovian excitatory and inhibitory conditioning In: Estes, W. K. (ed.), Handbook of learning and cognitive processes, 2 Hillsdale, N.J.: Erlbaum.

[41] Rescorla, R. A., & Cunningham, C. (1979). Spatial contiguity facilitates Pavlovian second-order conditioning. Journal of Experimental Psychology: Animal Behavior Processes, 5, 152–161. DOI: 10.1037/0097-7403.5.2.152 528884

[42] Rescorla, R. A., & Furrow, D. R. (1977). Stimulus similarity as a determinant of Pavlovian conditioning. Journal of Experimental Psychology: Animal Behavior Processes, 3, 203–215. DOI: 10.1037/0097-7403.3.3.203 881612

[43] Rescorla, R. A., & Holland, P. C. (1976). Some behavioral approaches to the study of learning In: Bennett, E., & Rozenzweig, M. R. (eds.), Neural mechanisms of learning and memory. Cambridge, Massachusetts: M.I.T. Press.

[44] Rescorla, R. A., & Wagner, A. R. (1972). A theory of Pavlovian conditioning: Variations in the effectiveness of reinforcement and nonreinforcement In: Black, A. H., & Prokasy, W. F. (eds.), Classical conditioning, 2 New York: Appleton.

[45] Revusky, S. (1971). The role of interference in association over a delay In: Honig, W. K., & James, P. H. R. (eds.), Animal memory, New York: Academic Press DOI: 10.1016/B978-0-12-355050-7.50009-6

[46] Revusky, S. (1977). The concurrent interference approach to delay learning In: Barker, L. M., Best, M. R., & Domjan, M. (eds.), Learning mechanisms in food selection. Waco, Texas: Baylor University Press.

[47] Revusky, S., & Garcia, J. (1970). Learned associations over long delays In: Bower, G. H., & Spence, J. T. (eds.), Psychology of learning and motivation: Advances in research and theory, 4 New York: Academic Press DOI: 10.1016/S0079-7421(08)60429-6

[48] Rozin, P. (1977). The significance of learning mechanisms in food selection: Some biology, psychology and sociology of science In: Barker, L. M., Best, M. R., & Domjan, M. (eds.), Learning mechanisms in food selection. Waco, Texas: Baylor University Press.

[49] Rudy, J. W., Iwens, S. J., & Best, P. J. (1977). Pairing novel exteroceptive cues and illness reduces illness-induced taste aversions. Journal of Experimental Psychology: Animal Behavior Processes, 3, 14–25. DOI: 10.1037/0097-7403.3.1.14 845542

[50] Seligman, M. E. P. (1968). Chronic fear produced by unpredictable electric shock. Journal of Comparative and Physiological Psychology, 66, 402–411. DOI: 10.1037/h0026355 5749134

[51] Seligman, M. E. P. (1969). Control group and conditioning: A comment on operationism. Psychological Review, 76, 484–491. DOI: 10.1037/h0028111

[52] Seligman, M. E. P., & Hager, J. L. (1972). Biological boundaries of learning. New York: Appleton.

[53] Seligman, M. E. P., Maier, S. F., & Solomon, R. L. (1971). Unpredictable and uncontrollable aversive events In: Brush, P. R. (ed.), Aversive conditioning and learning. New York: Academic Press DOI: 10.1016/B978-0-12-137950-6.50011-0

[54] Smith, E. R., & Miller, F. D. (1978). Limits on perception of cognitive processes: A reply to Nisbett and Wilson. Psychological Review, 85, 355–362. DOI: 10.1037/0033-295X.85.4.355

[55] Taylor, S. E., & Fiske, S. T. (1978). Salience, attention, and attribution: Top of the head phenomena In: Berkowitz, L. (ed.), Advances in experimental social psychology, 11 New York: Academic Press DOI: 10.1016/S0065-2601(08)60009-X

[56] Testa, T. J. (1974). Causal relationships and the acquisition of avoidance responses. Psychological Review, 81, 491–505. DOI: 10.1037/h0037183

[57] Testa, T. J. (1975). Effects of similarity of location and temporal intensity pattern of conditioned and unconditioned stimuli on the acquisition of conditioned suppression in rats. Journal of Experimental Psychology: Animal Behavior Processes, 104, 114–121. DOI: 10.1037/0097-7403.1.2.114 1141817

[58] Wagner, A. R. (1969). Stimulus validity and stimulus selection in associative learning In: Mackintosh, N. J., & Honig, W. K. (eds.), Fundamental issues in associative learning. Halifax: Dalhousie University Press.

[59] Wagner, A. R. (1978). Expectancies and the priming of STM In: Hulse, S. H., Fowler, H., & Honig, W. K. (eds.), Cognitive processes in animal behavior. Hillsdale, N.J.: Erlbaum.

[60] Winograd, E. (1971). Some issues relating animal memory to human memory In: Honig, W. K., & James, P. H. R. (eds.), Animal memory. New York: Academic Press DOI: 10.1016/B978-0-12-355050-7.50012-6

[61] Zener, K. (1937). The significance of behavior accompanying conditioned salivary secretion for theories of the conditioned response. American Journal of Psychology, 50, 384–403. DOI: 10.2307/1416644

